# A Case of Congenital Idiopathic Enlargement of Extraocular Muscles

**DOI:** 10.7759/cureus.59496

**Published:** 2024-05-02

**Authors:** Takashi Negishi, Shintaro Nakao

**Affiliations:** 1 Department of Ophthalmology, Juntendo University, Tokyo, JPN; 2 Department of Ophthalmology, Faculty of Medicine, Juntendo University, Tokyo, JPN

**Keywords:** ophthalmoplegia, inferior rectus muscle, cat scan, extraocular muscles, congenital idiopathic enlargement

## Abstract

The purpose of this case report is to report a case of congenital idiopathic enlargement of extraocular muscles. A four-month-old girl showed limitation of adduction and supraduction in the right eye. A computerized axial tomography (CAT) scan revealed hypertrophy of the lateral rectus muscle and inferior rectus muscle of the right eye. Thyroid hormone and antibody levels were normal. No inflammatory findings on magnetic resonance imaging (MRI). A traction test under general anesthesia revealed a strong limitation of supraduction and a mild limitation of adduction. Therefore, the inferior rectus muscle was recessed 4.5 mm at the age of six months. A partial biopsy of the inferior rectus showed no inflammatory cell infiltration. After the first surgery, the patient's limitation of supraduction improved, but the limitation of adduction persisted. So, a 5 mm recession of the right lateral rectus muscle was added at one year and one month. However, the hypertropia of the sound eye became stronger after treatment of amblyopia. Because of the strong limitation of supraduction, tenotomy of the inferior rectus was performed at the age of six years. Postoperatively, no impairment of infraduction occurred, and the limitation of supraduction was mildly improved. Since the findings on MRI were not changed through our observation period, we concluded that the patient had idiopathic external ophthalmoplegia.

## Introduction

The differential diagnosis for enlargement of extraocular muscles includes thyroid eye disease, hemangioma, lymphoma, leukemia infiltration, pseudotumor, myositis, sarcoidosis, and congenital fibrosis of the extraocular muscles (CFEOM) [1]. Congenital idiopathic enlargement of extraocular muscles (CIEOM) is a rare condition characterized by unilateral or bilateral enlargement and dysfunction of the extraocular muscles, often presenting in early childhood. The underlying etiology is unclear but may relate to aberrant innervation or primary muscle pathology. First described in the literature in the 1990s as "unilateral enlargement of the extraocular muscles," CIEOM can cause restrictive ophthalmoplegia and strabismus [2,3]. Reported cases demonstrate enlargement on imaging with sparing of the tendons and no evidence of inflammation, infiltration, or neoplastic process on biopsy [1,2]. Key considerations mentioned in the search results include thyroid eye disease, orbital inflammation, vascular anomalies, infections, metastasis, and restrictive strabismus syndromes [3,4]. A thorough evaluation is critical to rule out masquerading disorders. CIEOM often presents under the age of 10 years with variable patterns of dysmotility [5]. The lateral rectus is most commonly involved [5]. CIEOM is an important albeit uncommon cause of restrictive strabismus in pediatric patients [5]. We experienced a case of CIEOM without any other symptoms. This paper presents a case of unilateral extraocular muscle enlargement and reviews the literature on diagnostic considerations for CIEOM.

## Case presentation

**Figure 1:**
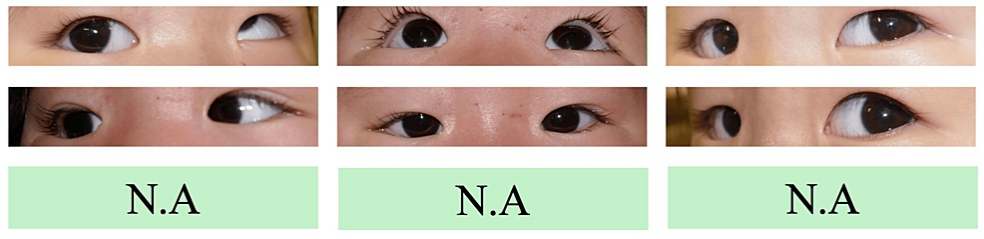
Image of the eye at the first visit (four months). Limitation of supraduction of the right eye is observed.

**Figure 2:**
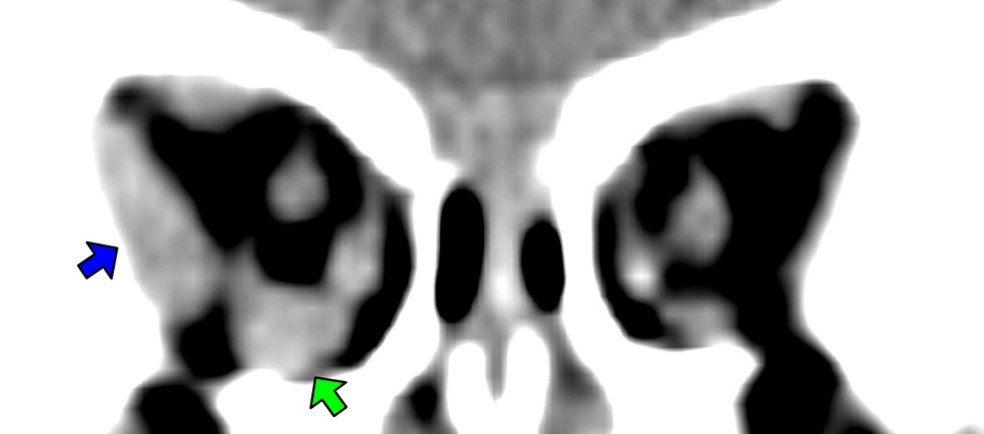
CAT scan image at the first visit. A coronal view of the CT orbit revealed enlargement of the lateral rectus and inferior rectus. The blue arrow is the right lateral rectus muscle, and the green arrow is the right inferior rectus muscle. CT, computerized tomography; CAT, computerized axial tomography

**Figure 3:**
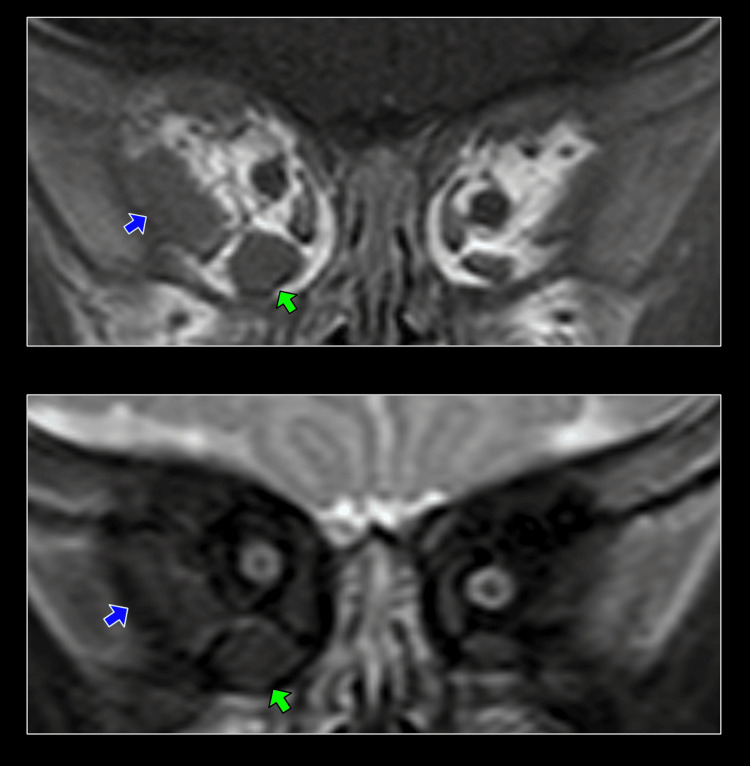
MRI and Short Tau Inversion Recovery (STIR) image at the first visit. The T1 phase of the MRI orbit revealed enlargement of the lateral rectus and inferior rectus. (Upper images) The blue arrow is the right lateral rectus muscle, and the green arrow is the right inferior rectus muscle. There are no inflammatory findings on the lower images (STIR images). The blue arrow is the right lateral rectus muscle, and the green arrow is the right inferior rectus muscle. MRI, magnetic resonance imaging

A four-month-old girl visited us because of difficulty with eye contact. This case was born at 40 weeks gestation with a normal delivery weight of 3,226 g. This case had no developmental delay and no abnormal findings in either eye. She showed limitation of adduction and supraduction in the right eye (Figure 1). Although we suspected a defect of the superior rectus in the right eye, a computerized axial tomography (CAT) scan revealed hypertrophy of the lateral rectus muscle and inferior rectus muscle of the right eye (Figure 2). Thyroid hormone and antibody levels were normal, and there were no inflammatory findings on the magnetic resonance imaging (MRI) (Figure 3). Muscle biopsy and strabismus surgery were performed at the age of six months. 

**Figure 4:**
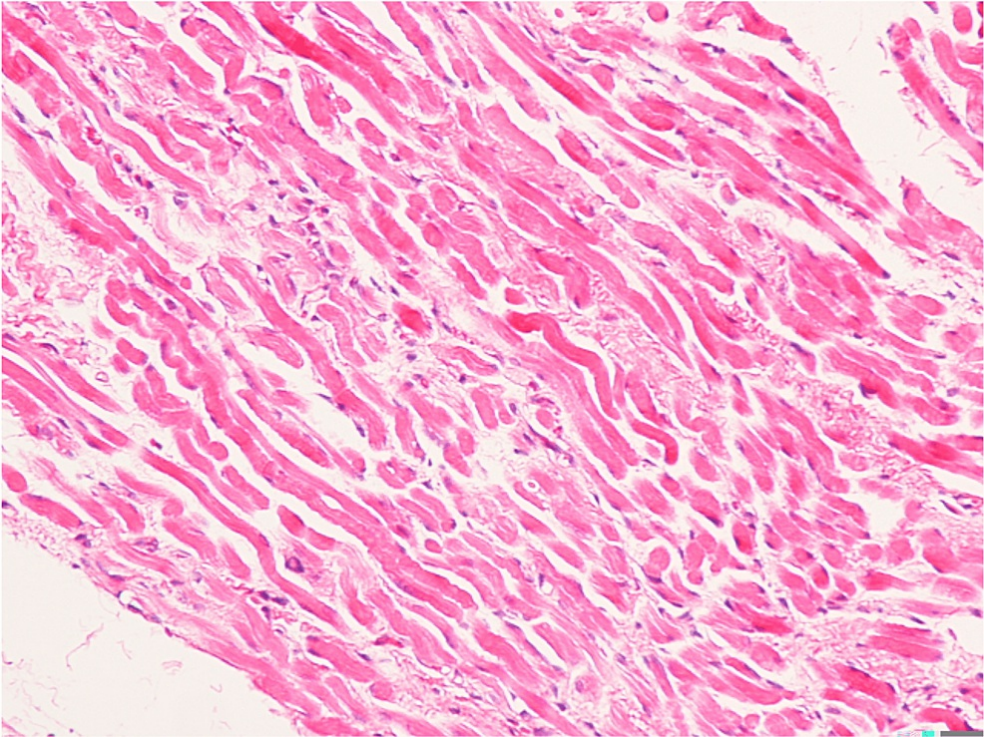
Pathological image of the inferior rectus muscle (hematoxylin and eosin stain). No inflammatory cell infiltration or neoplastic changes were found.

**Figure 5:**
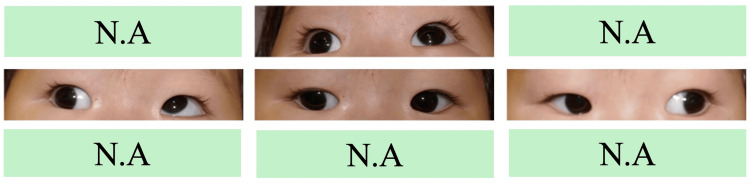
Nine gaze photographs before the second surgery. The limitation of adduction of the right eye became obvious.

At the first surgery, a force duction test under general anesthesia revealed a strong limitation of supraduction and mild limitation of adduction. Therefore, the inferior rectus muscle was recessed 4.5 mm without intervention onto the lateral rectus muscle. A partial biopsy of the inferior rectus muscle belly 12 mm posterior from the attachment was examined pathologically, but there was no inflammatory cell infiltration or neoplastic changes (Figure 4). After the first surgery, the patient's limitation of supraduction improved, but the limitation of adduction persisted and became obvious (Figure 5).

**Figure 6:**
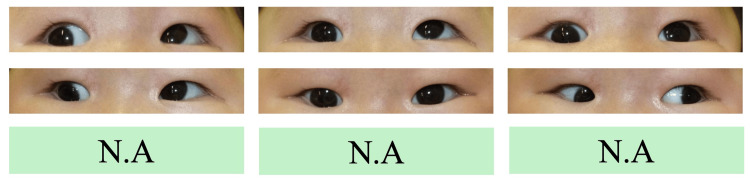
Nine gaze photographs one year after the second surgery. The limitation of supraduction of the right eye became obvious.

**Figure 7:**
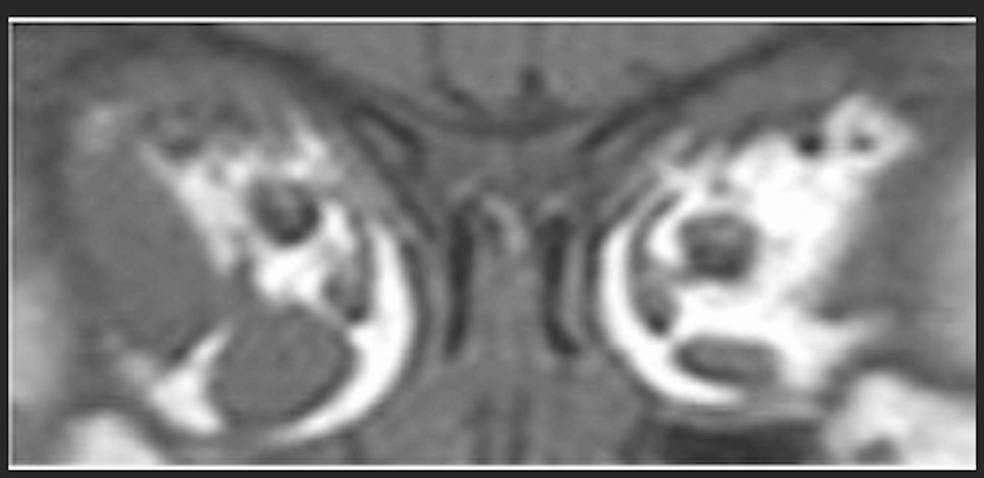
MRI findings one year after the second surgery. MRI findings were the same as the first MRI. MRI, magnetic resonance imaging

**Figure 8:**
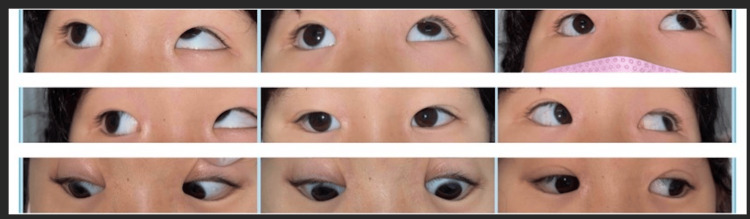
Nine gaze photographs just before the third surgery. Hypertropia of the left eye became noticeable.

**Figure 9:**
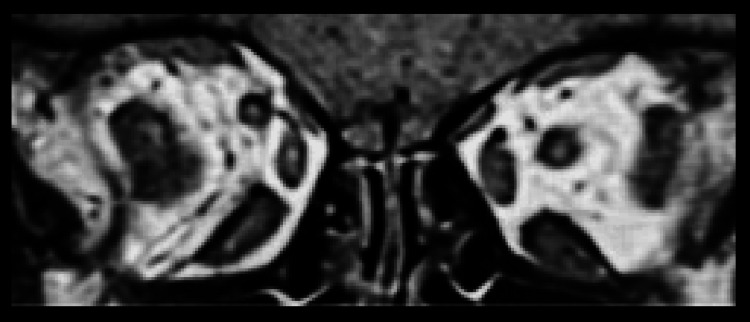
MRI findings just before the third surgery. MRI, magnetic resonance imaging

At the second surgery, a 5 mm recession of the right lateral rectus muscle was added at one year and one month of age. As the results of the second surgery, the head turn was improved and treatment for amblyopia was started (Figures 6-7). The patching in the left eye started at the first visit. By the last visit, the child could tolerate one to two hours of patching per day. By age four, his vision had developed to (1.2), so the patching was stopped at age six. After the patching on the left eye, the visual acuity of the right eye became finally (1.2) in each eye. However, hypertropia of the sound eye became noticeable, so additional surgery was performed at six years and five months (Figures 8-9).

**Figure 10:**
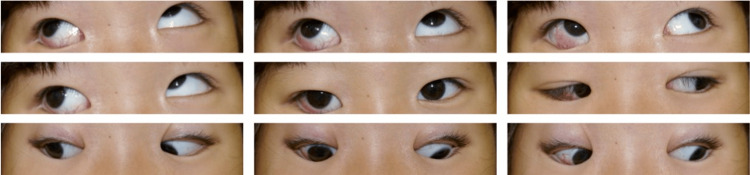
Nine gaze photographs three months after the third surgery.

During the third surgery, we found that the inferior rectus muscle was attached 5 mm from its original insertion, but the contraction was so strong that it was difficult to insert a strabismus hook. The attachment between the sclera and the inferior rectus muscle was removed as much as possible, and the inferior rectus muscle was encapsulated within Tenon’s capsule without sutures on the sclera. Postoperatively, no impairment of infraduction occurred, and the limitation of supraduction was mildly improved (Figure 10).

## Discussion

The initial presentation of difficulty with eye contact and specific limitations in adduction and supraduction led to a suspicion of superior rectus muscle defect [6]. However, a CAT scan revealed hypertrophy of the lateral and inferior rectus muscles. Additional inspection, including normal thyroid function, no inflammatory signs in MRI, and pathological findings suggested a rare case of CIEOM. This condition remains poorly understood due to its rarity and the idiopathic nature of its presentation [7]. Similar to the case presented by Modi et al., our case involved a young patient with significant unilateral hypertrophy of the lateral and inferior rectus muscles without any identifiable cause, despite comprehensive diagnostic evaluations [2].

The differential diagnosis for unilateral or bilateral enlargement of the extraocular muscles is broad, encompassing thyroid eye disease, orbital inflammation, and several neoplastic and vascular anomalies, among others [8-10]. However, the absence of inflammatory, neoplastic, or fibrotic changes on biopsy, normal thyroid function, and lack of response to typical treatments for these conditions differentiate CIEOM from these other diagnoses. The literature review by Modi et al. underscores the exceptional rarity of this presentation and the importance of distinguishing it from thyroid eye disease and other causes of extraocular muscle enlargement that can have significant systemic implications [2].

Our case contributes to the limited but growing body of evidence, suggesting that CIEOM may represent a distinct clinical entity. The etiology of CIEOM remains speculative [2]. However, the consistent lack of inflammatory or neoplastic findings on muscle biopsy across reported cases, including ours, suggests noninflammatory pathogenesis [2,5]. The pathology findings in our case, showing normal muscle architecture without evidence of cellular infiltration or fibrosis, are in line with those described by Modi et al., further supporting the idiopathic nature of this condition [2].

The management of CIEOM poses a significant challenge due to its rarity and the lack of established treatment guidelines [2,10]. In our case, as well as in the case reported by Modi et al., surgical intervention was pursued to address the restrictive strabismus, with partial success [2]. In our case, because the supraduction disorder was very strong, and strabismic amblyopia was also a concern, surgery was performed before quantification of the strabismic angle. Otherwise, the need for multiple surgeries in our patients highlights the complexity of managing this condition and the need for individualized treatment plans.

## Conclusions

Our case report highlights the complexity of diagnosing and managing ocular motility disorders in young children. This patient was diagnosed with *congenital idiopathic extraocular muscle enlargement *based on MRI findings, laboratory data, and pathological examinations at the time. The absence of inflammatory signs and normal thyroid function in our patient further narrowed the focus of our investigation, underscoring the importance of a comprehensive and systematic approach in the evaluation of pediatric strabismus cases with atypical presentations.
